# Temporal patterns of energy intake and physical activity and cross-sectional associations with body weight status in children and adolescents: results from the Portuguese National Food, Nutrition and Physical Activity Survey 2015–2016

**DOI:** 10.1017/S0007114524002861

**Published:** 2024-12-28

**Authors:** Sofia Cardoso, Inês Sanches, Daniela Correia, Sofia Vilela

**Affiliations:** 1 EPIUnit ITR - Instituto de Saúde Pública, Universidade do Porto, Rua das Taipas, n° 135, 4050-600 Porto, Portugal; 2 Faculdade de Medicina, Universidade do Porto, Alameda Prof. Hernâni Monteiro, 4200-319, Porto, Portugal; 3 Departamento de Saúde Pública e Ciências Forenses e Educação Médica, Faculdade de Medicina, Universidade do Porto, Alameda Prof. Hernâni Monteiro, 4200-319 Porto, Portugal

**Keywords:** Feeding behaviour, Eating patterns, Energy intake, Chrononutrition, Physical activity, Paediatric obesity

## Abstract

Temporal energy intake (EI) and physical activity (PA) patterns may be associated with obesity. We aimed to derive and characterise temporal EI and PA patterns, and assess their cross-sectional association with weight status, in 6-to-14-year-old Portuguese participants of the National Food, Nutrition and Physical Activity Survey 2015–2016. We extracted times and EI of all eating occasions from two 1-d food diaries/24-h recalls, while types and times of PA from 4-d PA diaries. We derived EI patterns (*n* 714) and PA patterns (*n* 595), using, respectively, a hierarchical and K-means cluster analysis, considering the average proportion of total daily EI (%TEI) and PA intensity (%TPA), within each 2-h interval across the 24-h day. Patterns were labelled based on the 2-h intervals of %TEI/TPA peaks. We assessed the association between patterns and overweight or obesity (BMI z-score ≥ +1 sd) using adjusted logistic regressions (OR (95 % CI)). Three EI patterns emerged: 1 – ‘Early afternoon and early evening’; 2 – ‘Early afternoon and late evening’; and 3 – ‘Late morning, early and mid-afternoon and late evening’. EI Pattern 3 *v*. Pattern 1 was negatively associated with overweight or obesity (0·49 (0·26, 0·92)). PA Pattern 1 – ‘Late morning, mid-afternoon and early evening’ *v*. Pattern 2 – ‘Late afternoon’, was not associated with weight status (0·95 (0·65, 1·38)). A daily EI pattern with more and even %TEI peaks at earlier daytime periods, rather than fewer and higher, may be negatively associated with overweight or obesity amongst this population whereas the identified PA patterns might have no relationship.

The obesity epidemic constitutes one of the major public health concerns of the 21st century^(1)^. School-age children and adolescents experience important physiological, anatomical and behavioural changes. Paediatric obesity and related behaviours, including poor dietary and physical activity (PA) habits, are especially concerning given its tracking into adulthood^(1,2)^. Their cumulative health impacts are associated with an increased risk of chronic diseases and disability-adjusted life years^(1,2)^. Dietary intake and PA are crucial modifiable risk factors, yet prevention strategies have mostly focused on diet composition, PA intensity and frequency^(3)^.

Chronobiology studies suggest that the alignment of an individual’s internal circadian clock system with the 24-h light/dark cycle is crucial to metabolism homeostasis and weight regulation^(4)^. Biological clocks govern and are synchronised by physiological and behavioural rhythms, including sleeping and feeding^(5)^. If food intake and locomotor activity occur later than the favoured daytime/light period^(5)^, internal rhythmicity is affected^(6)^. Age, sex, family practices, school and extracurricular activity schedules and other dietary and activity behaviours also influence food intake and PA timings^(7)^.

Later eating patterns, marked by concentrating meals or energy intake (EI) during the evening/nighttime instead of earlier times, may disrupt appetite and nutrient and energy metabolism, increasing the risk of obesity^(5)^. Different measures (mealtimes and/or EI) and criteria to define ‘later’ eating rhythms/patterns (periods or time cut-offs) have contributed to their inconsistent association with higher adiposity in adults^(8,9)^ and children^(10)^.

Although physiological responses to exercise may vary with the time of day of its practice^(11)^, few studies in adults found an association between PA patterns, based on the times and intensity of all activities, and weight status^(9,12)^.

Notwithstanding the potential effect of feeding and PA rhythms in modulating the risk of paediatric obesity, evidence of the relationship between the temporal distribution of EI and PA with adiposity in paediatric age remains uncertain and scarce.

Hence, the present study aimed to derive and characterise daily temporal EI and PA patterns, based on a cluster analysis of the EI and PA intensity within each 2-h interval across the 24-h day, and to assess their cross-sectional association with weight status, based on BMI, in children and adolescents. Considering biological plausibility and current studie’s limitations, we hypothesise that at least two EI and PA clusters will emerge, and the one with a later *v*. earlier distribution will be positively associated with worse weight status.

## Methods

### Study design and participants

The present study used data from the Portuguese National Food, Nutrition and Physical Activity Survey 2015–2016 (IAN-AF)^(13,14)^. Briefly, this cross-sectional survey used the National Health Registry as a sampling frame and applied a multi-stage sampling to obtain a representative sample of the general Portuguese population aged between 3 months and 84 years. First, in each of the seven Portuguese NUTS II geographic regions, health units were randomly selected, weighted by the number of registered health users. Then, within each unit, individuals were randomly selected, with a fixed number by sex and age group. A total of 5811 participants completed both interviews of the survey, of whom 1327 were aged 3 months to nine years and 632 were aged 10–17 years.

For the present study, the inclusion criteria were school-age participants aged between 6 and 14 years, with two valid dietary intake assessments and height and weight measurements (*n* 714). For the EI patterns analyses, we included 714 participants. For the analyses concerning PA patterns, additional inclusion criteria were to have a minimum of three PA diaries, with at least 12 h of reported activities, on two weekdays and one weekend day, resulting in a sub-sample of 595 participants. Considering that PA diaries were only applied amongst 6- to 14-year-olds in the IAN-AF and to ensure a homogenous age range for the present study, we selected this inclusion criterion, with no requirement regarding the proportions of children (6–9 years) and adolescents (10–14 years).

### Data collection

The variables of interest of this study were extracted from the IAN-AF dataset or obtained through additional calculations and categorisations.

In the IAN-AF, data collection was conducted between October 2015 and September 2016, covering the annual four seasons and every day of the week, through two personal computer-assisted interviews (8–15 d apart) performed by trained interviewers, using the ‘You eAT&Move’ platform developed for the survey^(13,14)^. Data on dietary intake, PA, health and sociodemographic variables were collected.

In our study, to capture the temporal EI and PA patterns, we analysed the distribution of total daily EI (TEI) and total daily PA intensity (TPA) at each 2-h interval across the 24-h day, as analysed in previous studies^(15–17)^. We adopted this approach to avoid the limitations of using meal definitions or arbitrary clock time cut-offs to define earlier/later patterns. The 2-h intervals provided a feasible number of clusters compared to 1-h interval, and sufficient detail to capture distribution differences, compared to longer intervals. These intervals also aligned with typical Portuguese frequency and times of meals^(18)^, as well as with parents’ work and participants’ school schedules.

All clock times are in local time and 24-h format (h:min).

### Dietary intake assessment

In the IAN-AF, dietary intake was assessed according to European guidelines^(19)^ through two non-consecutive 1-d food diaries filled by the children’s main caregiver and through two non-consecutive 24-h recalls administered to adolescents accompanied by caregivers. On each day, from 00:00 to 23:59, participants had to report the wake-up time, types and quantities of every consumed food and beverage (including recipes) per eating occasion, plus their respective place and time. Eating occasions included three main meals (breakfast, lunch and dinner) which could be reported once daily, and all snacks consumed before and after each of the main meals. All data were computerised using the ‘eAT24’ software^(20)^ which was linked with FoodEX2^(21)^ and an extended version of the Portuguese Food Composition Table that included recipes^(22)^ to estimate energy and nutrient intake.

### Temporal energy intake patterns

To obtain the temporal EI patterns, one of the independent variables of interest in our study, we performed three steps: (1) for each participant and each dietary intake report, we extracted the clock times and EI of each eating occasion and the TEI (in kilojoules (kJ)); (2) for each participant, using the two reports, we computed the daily average proportion of TEI (%TEI) ingested at each 2-h interval across the 24-h day (00:00–23:59); and (3) considering these data, we performed a hierarchical cluster analysis with the complete linkage method^(23)^. This method does not allow the user to predefine an expected number of clusters; instead, it generates a cluster tree by sequentially agglomerating similar data points into larger groups (clusters). We trimmed the cluster tree based on the Dunn Index^(24)^, obtaining three clusters (i.e. patterns).

### Diet-related covariates

We considered the below-cited dietary-related covariates to characterise participants and/or assess as potential confounders or effect modifiers in the logistic regression analysis.

Participants’ diet quality was assessed using a previously developed and validated index in Portuguese children, termed the healthy eating index^(25,26)^. This index indicates adherence to a healthier diet and was based on the WHO’s paediatric dietary recommendations^(27)^ for nine food groups, including five considered *‘healthier’: cereals and potatoes*; *dairy products*; *fruit and vegetables*, *including legumes*; and *white meat, fish and eggs*; and four *‘less healthy’: red meat and processed meat*; *salty snacks*; *sugar-sweetened beverages*; *sugar and honey*; and *sweets.* For each group, the average daily consumption was calculated (g/d), and respective quartiles were obtained, for children and adolescents. Each quartile was scored between 1 and 4 points, in an ascending order for healthier food groups and in a descending order for the less healthy. Given the high prevalence of non-consumers, we re-categorised the consumption of *salty snacks* and *sugar and honey* as *No* (0 g/d) or *Yes* (> 0 g/d), which scored 2 and 1 points, respectively. The summed-up score ranged from 9 to 32 points.

We estimated each participant’s average TEI (kJ/d) and respective contributions of proteins, carbohydrates and fats (%TEI), plus dietary fibre intake (g/d).

We computed the daily average number of all reported eating occasions (*n*/d) and categorised breakfast-skipping as *No* (reported on both days) or *Yes* (reported on ≤ 1 d).

To further explore the temporal distribution of the patterns, we calculated the daily average clock times of the following parameters: (i) breakfast, lunch and dinner; (ii) first and last EI occasions (not necessarily breakfast and dinner); (iii) ‘eating midpoint’, which is the midpoint of the eating window ((clock time of last EI occasion – clock time of first EI occasion)/2)^(28)^; and (iv) achievement of 25, 50 and 75 % of TEI, which represent the cumulative distribution of TEI by quartiles.

Furthermore, we categorised participants based on the type of days of their dietary intake reports. Most had both reports on weekdays (*n* 515, 72 %); 24 % (*n* 169) had one on a weekday and one on a weekend day, while 4% (*n* 30) had both reports on weekends.

### Physical activity assessment

In the IAN-AF, adolescents and children’s main caregivers were asked to fill a 4-d PA diary, adapted from Bouchard’s 3-d activity record^(29)^, over two consecutive weekdays and two weekend days. Briefly, in a logbook, they had to describe the main activity performed every 15-min interval across 24-h (00:00–23:59). Using the ‘Move’ module^(13,14)^, the PA intensity of each activity was estimated by multiplying the respective metabolic equivalent of task (MET) value by the time spent in the activity^(30,31)^. The TPA, in MET-h/d, corresponded to the sum of the PA intensity of all daily activities, excluding sleep. Additionally, all participants were asked about PA behaviours.

### Temporal physical activity patterns

To obtain temporal PA patterns, the second independent variable of interest in our study, we performed four steps: (1) for each day of the PA diary of each participant, we extracted the start and end times of each reported activity, the respective PA intensity (MET), and the TPA; (2) we assigned a unique clock time to each activity (excluding sleep), correspondent to the halfway between its start and end times (e.g. the activity ‘watching television while seated’ reported between 14:00 and 15:30 was assigned 14:45); (3) for each participant, considering the total number of PA diaries, we computed their daily average proportion of TPA (%TPA) expended at each 2-h interval across the 24-h day; and (4) considering these data, we performed a k-means cluster analysis^(32)^. This clustering method produces a partition of the data into a particular number of groups, while decreasing the variability within clusters and increasing the variability between clusters^(32)^. Two to ten clusters were tested, and the best partition of data was evaluated based on a set of thirty different indices^(33)^, resulting in two clusters.

### Physical activity-related covariates

We considered a set of PA-related covariates. The practice of regular extracurricular structured PA (*No/Yes*) was reported by children’s parents and by adolescents, for all participants.

For the sub-sample with PA diaries, we considered the daily average TPA (MET-h/d) of all reported PA diaries, and the daily average time spent (h/d) on PA with a moderate-to-vigorous intensity (MET ≥ 3) and in sedentary behaviour (MET ≤ 1·5)^(34)^. To further explore the temporal distribution of PA we calculated the daily average clock times of the following parameters using PA diaries: (i) first and last reported activities (excluding sleep), (ii) the midpoint of the PA window, and (iii) the achievement of 25, 50 and 75 % of TPA.

### Anthropometric assessment

Anthropometry was performed by trained staff according to standard procedures^(35)^ without shoes and with light clothing. Weight was measured to the closest 0·1 kg, using a digital scale (SECA® 813), and height was measured to 0·1 cm, using a portable wall stadiometer (SECA® 213). Sex-specific BMI-for-age z-scores (zBMI) were calculated using specific software and categorised according to the WHO criteria as underweight (< –2 sd), normal weight (–2 ≤ sd < +1), overweight (+1 ≤ sd ≤ +2) or obesity (> +2 sd)^(36)^.

### Body weight status

Participants’ body weight status based on the WHO’s zBMI categories constituted our study’s dependent variable (outcome). Within our sample (*n* 714), the prevalences of underweight, normal weight, overweight and obesity were, respectively: 1·3, 60·5, 25·4 and 12·9 %. Given the low prevalence of underweight, and previous studies usually aggregate categories, we re-categorised weight status as *underweight or normal weight* (zBMI < +1 sd) and *overweight or obesity* (zBMI ≥ +1 sd).

### Sociodemographic covariates

We considered participants’ sex, age (as a continuous variable), age group (*children* or *adolescents*), and the reported household size categorised as *3*, *4* or *> 4 living members*. Maternal current working status was self-reported and categorised as *employed* or *unemployed or other* (including student/retired/disabled). Parental education level was defined by the parent reporting the highest level of schooling completed, and was re-categorised as *secondary or lower* (none to secondary level, corresponding to 0–12 years) or *tertiary education* (post-secondary level, corresponding to > 12 years).

### Sleep and chronotype covariates

Individuals’ wake-up and bedtimes and circadian clock systems are related to temporal PA and EI patterns^(7,37,38)^. Chronotype is a biological construct that reflects individual differences in the circadian system^(37,39)^. According to Roenneberg, an individual’s chronotype can be estimated using the Munich Chronotype Questionnaire, by determining the midpoint of sleep on free days, as halfway between sleep onset and sleep offset clock times on school-free days, and correcting it for sleep debt accumulated during school days. This originates the midpoint of sleep on free days corrected (MSFsc in h:min)^(37,39)^. Following these principles, we estimated participants’ chronotype for the sub-sample with PA diaries assuming weekends were free days, without interferences in sleep times, and that the reported sleep start and end times of the activity ‘sleep’ were proxies of sleep onset and end times^(38)^, respectively. For each participant, we extracted sleep start and end times on weekdays and weekend days, and calculated the respective sleep duration. We computed MSFsc (n=591) through a computation process detailed elsewhere^(38)^ that implied data imputation for missing sleep start times.

### Statistical analysis

We characterised the sample included in EI patterns analyses and the sub-sample of PA patterns analyses and compared the sub-sample with excluded individuals.

Continuous variables were summarised by the mean and sd if the distribution was considered normal through visually inspecting histograms and Q–Q plots or by the median and interquartile range. Differences were assessed by one-way ANOVA or Student’s *t* test or by the respective non-parametric tests Kruskal–Wallis or Independent–sample Mann–Whitney *U* test, appropriately. Categorical variables were summarised by frequency(*n*) and column proportions (%), and differences were assessed using Pearson’s *χ* ^2^ test.

The temporal EI patterns and PA patterns, derived by cluster analysis, were characterised separately using a plot depicting the daily average %TEI and %TPA at each 2-h interval across the 24-h day (00:00–23:59). To supplement these, we summarised and compared the %TEI at each 2-h interval between EI patterns and the%TPA between PA patterns. We labelled each pattern based on the 2-h intervals at which the highest values of %TEI/TPA (‘peaks’) occurred. First, within the 24-h day, we identified one nighttime period (22:00–05:59) and three daytime periods, morning (06:00–11:59), afternoon (12:00–17:59) and evening (18:00–21:59), which were further split into 2-h periods (early, mid and late). Each pattern could have more than one peak if there were similarly high values of %TEI/TPA at different intervals, which we compared using related samples Friedman’s two-way ANOVA by ranks.

Participant’s characteristics were summarised and compared according to their EI and PA Patterns, separately.

The associations of EI and PA patterns (Pattern 1 as the reference category) with weight status (*overweight or obesity* as the response) were assessed separately by logistic regression analysis. For the independent variable EI patterns, we fitted four nested models: a crude model (model 1) and adjusted models for sex, age and parental education (model 2), healthy eating index score (model 3) and structured PA practice (model 4). For PA patterns, we conducted models 1, 2 and 3, plus models adjusted for the time spent in sedentary behaviour (model 4) and MSFsc (model 5). Results are expressed as OR and the respective 95 % CI. There was no significant interaction effect for age, age group or MSFsc (assessed only for PA patterns).

As supplementary analyses, we compared PA-related covariates between EI patterns for the sub-sample included in PA Patterns, and explored the potential role of the type of day and circannual periods^(38,40)^ of the dietary intake and PA reports on the EI and PA Patterns. First, we assessed the association between the types of days of the dietary intake reports and EI patterns. For participants with reports on weekdays and weekends (*n* 169), we compared daily average clock times of EI parameters computed with *v.* without weighing for the type of day, as we applied in this study. Second, we categorised each participant’s period of dietary intake reports and PA diaries, as *summer school holidays* if ≥ 1 day occurred between 9 June and 15 September 2016 or *other*, if none did. Then, we assessed the associations of the period of dietary intake/PA reports with EI/PA patterns and weight status.

EI and PA patterns were derived in R statistical computing programme, version 3.4.1 (R Foundation for Statistic Computing, Austria, 2010), and the remaining analyses were conducted in Statistical Package for the Social Sciences, version 26.0 (IBM SPSS Statistics for Windows, IBM Corp.).

## Results

### Participant characteristics

The sample included in EI patterns (*n* 714, 51 % girls) had a mean age of 10 (sd = 2·7) years, zBMI of 0·70 (sd = 1·11), and 38·2 % had overweight or obesity. Most practised structured PA (61 %) had employed mothers (82 %) and parents with a secondary or lower level of education (64 %). The sub-sample included in PA patterns (*n* 595) *v*. EI patterns had similar characteristics. However, compared with participants without PA diaries (*n* 119), the proportion of overweight or obesity was lower (36 *v*. 47 %, *P* = 0·03) (Table 1).

**Table 1.** Participants’ characteristics according to the sample of temporal energy intake (EI) patterns (*n* 714), sub-sample of temporal physical activity (PA) patterns (*n* 595) and comparison with excluded individuals (*n* 119) (Numbers and percentages; mean values and standard deviations)

	Sample of EI patterns		Sub-sample of PA patterns		Excluded individuals		
	*n* 714		*n* 595		*n* 119		
	*n* Mean*	% sd *	*n* Mean*	% sd *	*n* Mean*	% sd *	*P* †
Sex							0·50
Female	362	50·7	305	51·3	57	47·9	
Male	352	49·3	290	48·7	62	52·1	
Age, years	10·0	2·69	10·1	2·68	9·8	2·69	0·29
Age group							0·36
Children	297	41·6	243	40·8	54	45·4	
Adolescents	417	58·4	352	59·2	65	54·6	
Household size‡							0·95
2–3 members	192	29·6	158	29·3	34	30·9	
4 members	361	55·6	301	55·8	60	54·5	
> 4 members	96	14·8	80	14·8	16	14·5	
Parental education level‡							0·11
Secondary or lower	452	63·9	369	62·6	83	70·3	
Tertiary	255	36·1	220	37·4	35	29·7	
Maternal age, years‡	40·3	5·31	40·5	5·00	39·3	5·99	0·060
Maternal working status‡							0·34
Employed	578	81·8	486	82·4	92	78·6	
Unemployed or other	129	18·2	104	17·6	25	21·4	
z-score BMI (zBMI), sd	0·70	1·11	0·68	1·10	0·83	1·17	0·16
Weight status							0·030
Underweight or normal weight	441	61·8	378	63·5	63	52·9	
Overweight or obesity	273	38·2	217	36·5	56	47·1	
Temporal EI patterns							0·005
EI Pattern 1 – Early afternoon and early evening	144	20·2	107	18	37	31·1	
EI Pattern 2 – Early afternoon and late evening	498	69·7	426	71·6	72	60·5	
EI Pattern 3 – Late morning, early and mid-afternoon and late evening	72	10·1	62	10·4	10	8·4	
Healthy eating index score, points	20·4	3·39	20·4	3·40	20·7	3·36	0·44
TEI, kJ	7958	2169·0	7941	2164·0	8037	2202·5	0·67
Temporal PA patterns	NA	NA	595		NA	NA	
PA Pattern 1 – Late morning, mid-afternoon and early evening	NA	NA	381	64·0	NA	NA	
PA Pattern 2 – Late afternoon	NA	NA	214	36·0	NA	NA	
Structured PA‡							0·78
No	271	38·8	225	38·6	46	38·6	
Yes	427	61·2	358	61·4	69	61·4	

TEI, total daily energy intake; NA, not applicable, because there was no data available.

* Data are presented as frequency (*n*) and column proportions (%) for categorical variables and as mean and standard deviation (sd) for continuous variables.

† *P*-value for Pearson’s *χ* ^2^ test for categorical variables and Student’s *t* test for continuous variables conducted between the sub-sample PA patterns and excluded individuals.

‡ Based on *n* 616 for the sample of EI patterns, *n* 514 for the sub-sample of PA patterns and *n* 102 for excluded individuals, due to missing data. For categorical variables, the sum of each category did not add up to the *n* of each column due to missing data.

### Temporal energy intake patterns

**Figure 1.**
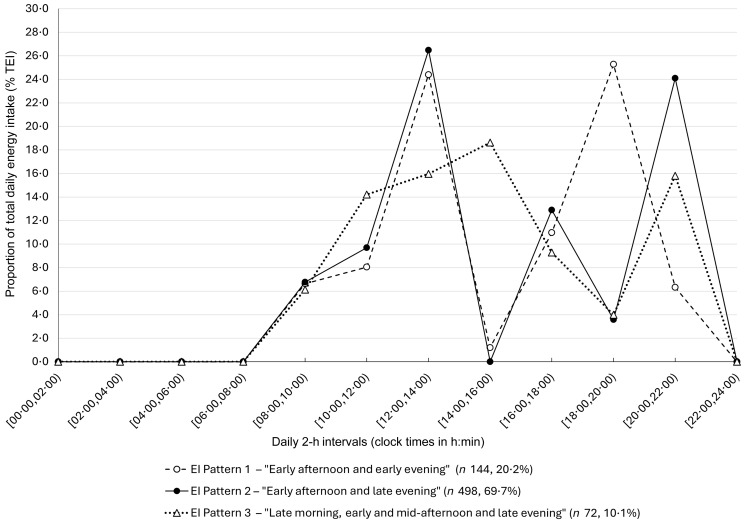
Daily average proportion of total daily energy intake (%TEI) ingested at each 2-h interval across the 24-h day (from 00:00 until 23:59), for the three identified temporal EI patterns (*n* 714). The median values of %TEI are presented due to the non-normal distribution of the variables. The bounds of each 2-h interval are in local clock times (h:min). EI Pattern 1 – ‘Early afternoon and early evening’ (*n* 144, 20·2 %) (white circles and dashed line). EI Pattern 2 – ‘Early afternoon and late evening’ (*n* 498, 69·7 %) (black circles and full line). EI Pattern 3 – ‘Late morning, early and mid-afternoon and late evening’ (*n* 72, 10·1 %) (triangle and dotted line).

Three temporal EI patterns were identified, whose distribution of %TEI across the 24-h day is presented in Fig. 1 and detailed in online Supplementary Table S1. EI Pattern 1 – ‘Early afternoon and early evening’ had two peaks providing around 25 % of TEI, at two 2-h intervals: 12:00–13:59 and 18:00–19:59. Pattern 2 – ‘Early afternoon and late evening’ was the most prevalent (70 % of the sample) and also had two %TEI peaks (24 and 26 %) at 12:00–13:59 and 20:00–21:59. Pattern 3 – ‘Late morning, early and mid-afternoon and late evening’ (10 %, *n* 72) had four similar %TEI peaks, ranging from 14 to 19 %; three occurred at 2-h intervals between 10:00 and 15:59 and the last at 20:00–21:59. Although participants with Pattern 3 presented later main meals, they achieved 50 % of TEI (15:00 *v*. 14:35 and 14:22, *P* = 0·009) and 75 % of TEI earlier than the other patterns (Table 2). Overweight or obesity was less prevalent in participants with Pattern 3 *v*. Patterns 1 and 2 (28 *v*. 44 and 38 %, *P* = 0·06) (Table 2).

**Table 2.** Participants’ characteristics according to temporal energy intake (EI) patterns (*n* 714) (Numbers and percentages; mean values and standard deviations; median values and interquartile ranges)

	EI Pattern 1 – Early afternoon and early evening		EI Pattern 2 – Early afternoon and late evening		EI Pattern 3 – Late morning, early and mid-afternoon and late evening		
	*n* 144		*n* 498		*n* 72		
	*n* mean or median*	% sd or IQR*	*n* mean or median*	% sd or IQR*	*n* mean or median*	% sd IQR*	*P* †
Sex							0·51
Female	67	46·5	259	52	36	50	
Male	77	53·5	239	48	36	50	
Age, years	10·0	2·88	9·93	2·64	10·6	2·56	0·18
Age group							0·13
Children	62	43·1	213	42·8	22	30·6	
Adolescents	82	56·9	285	57·2	50	69·4	
Maternal age, years‡	39·1	5·34	40·7	5·18	39·6	5·74	0·007
Parental education level							0·029
Secondary or lower	101	70·6	299	60·8	52	72·2	
Tertiary	42	29·4	193	39·2	20	27·8	
Maternal working status‡							0·46
Employed	119	82·6	404	82·3	55	76·4	
Unemployed or other	25	17·4	87	17·7	17	23·6	
zBMI, sd	0·93	1·14	0·67	1·11	0·49	1·01	0·01
Weight status							0·06
Underweight or normal weight	80	55·6	309	62·0	52	72·2	
Overweight or obesity	64	44·4	189	38·0	20	27·8	
Healthy eating index score, points	20·3	3·46	20·6	3·37	19·6	3·28	0·05
TEI, kJ	7920	2087·0	7950	2159·4	8083	2412·9	0·87
Protein, %TEI	17·3	3·27	17·1	3·32	16·3	3·47	0·11
Carbohydrates, %TEI	50·6	5·89	51·4	6·15	52·0	5·46	0·25
Free sugars, %TEI	12·4	6·07	12·1	5·97	14·1	6·75	0·04
Fibre, g/d§	14·2	5·91	15·4	6·56	14·3	6·14	0·25
Fat, %TEI	30·2	5·74	29·6	5·72	29·8	5·24	0·52
Trans fatty acids, %TEI§	0·43	0·26	0·43	0·29	0·45	0·40	0·49
Saturated fatty acids, %TEI	11·0	2·6	10·66	2·66	11·1	2·61	0·08
Daily frequency of eating occasions, *n*/d	6	1·1	6	1·3	6	1·4	0·97
Breakfast-skipping							0·61
No	130	90·3	462	92·8	66	91·7	
Yes	14	9·7	36	7·2	6	8·3	
Breakfast, h:min§	08:12	1:15	08:29	1:30	08:37	1:44	0·01
Lunch, h:min§	12:47	0:42	12:55	0:42	13:13	1:24	0·001
Dinner, h:min	19:30	0:48	20:17	0:45	20:25	0:48	< 0·001
First EI occasion, h:min§	08:15	1:21	08:30	1:32	08:31	1:42	0·56
25 % of TEI, h:min§	12:00	1:57	11:55	1:45	11:47	2:30	0·99
50 % of TEI, h:min§	15:00	2:11	14:35	2:42	14:22	2:06	0·009
75 % of TEI, h:min§	19:00	1:30	19:08	2:07	18:00	2:03	< 0·001
Last EI occasion, h:min§	20:45	1:30	21:00	1:00	21:00	1:51	0·11
Eating midpoint, h:min§ ^,║^	14:37	1:18	14:45	1:04	14:49	1:22	0·19
Structured PA‡							0·23
No	61	43·3	179	36·8	31	44·3	
Yes	80	56·7	308	63·2	39	55·7	

IQR, interquartile range; yzBMI, z-score BMI; PA, physical activity; TEI, total daily energy intake.

* Data are presented as frequency (*n*) and column proportions (%) for categorical variables, as mean and standard deviation (sd) for continuous variables or as the median and interquartile range, if these were non-normally distributed.

† *P*-values for Pearson’s *χ* ^2^ test for categorical variables and one-way ANOVA or Kruskal–Wallis test for continuous variables.

‡ Based on *n* 616, due to missing data. For categorical variables, the sum of each category did not add up to the *n* of each column due to missing data.

§ Variables for which median (IQR) and *P*-value for Kruskal–Wallis test are presented.

║ Calculated as (clock time of last EI occasion – clock time of first EI occasion)/2.

Online Supplementary Table S2, which compared PA-related covariates between EI patterns for the sub-sample of PA diaries (*n* 595), showed that participants with EI Pattern 1 *v*. Pattern 2 had earlier sleep start and end times on weekdays. EI patterns were not associated with participants’ type of days of dietary reports (*χ* ^2^ test *P* = 0·95). Additionally, online Supplementary Table S3 showed that most EI parameters calculated with or without weighting for weekdays and weekends were similar (*n* 169). EI Pattern 2 *v*. Pattern 1 was associated with having dietary intake reports during summer school holidays compared to other periods (online Supplementary Table S4).

### Temporal physical activity patterns

**Figure 2.**
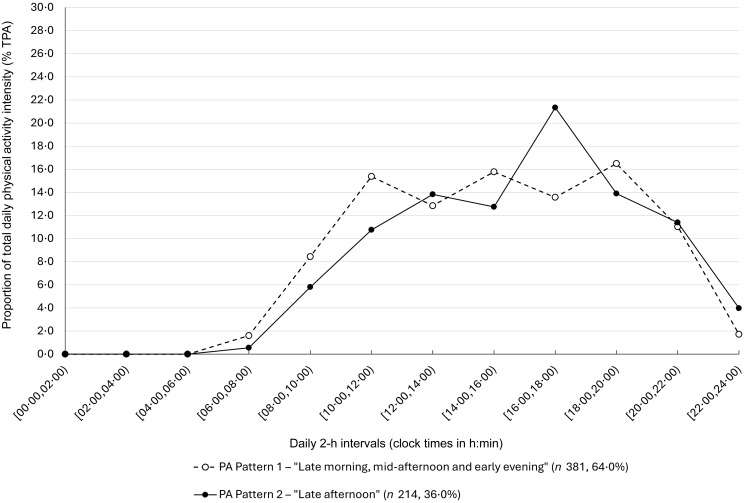
Daily average proportion of total daily physical activity intensity (%TPA) expended at each 2-h interval across the 24-h day (from 00:00 until 23:59), for the two identified temporal PA patterns (*n* 595). The median values of %TPA are presented due to the non-normal distribution of the variables. The bounds of each 2-h interval are in local clock times (h:min). PA Pattern 1 – ‘Late morning, mid-afternoon and early evening’ (*n* 381, 64·0 %) (white circles and dashed line). Pattern 2 – ‘Late afternoon’ (*n* 214, 36·0 %) (black circles and full line).

The daily distribution of %TPA of the two identified temporal PA patterns is depicted in Fig. 2 and detailed in online Supplementary Table S5. PA Pattern 1 – ‘Late morning, mid-afternoon and early evening’, was presented by most of the sub-sample (64 %) and had three peaks of %TPA, ranging from 15 to 16 %, at 10:00–11:59, 14:00–15:59 and 18:00–19:59. PA Pattern 2 – ‘Late afternoon’, had a single %TPA peak of 21 % at 16:00–17:59. Pattern 2 achieved quartiles of TPA at later clock times and had later sleep start and end times (Table 3). Participants who presented PA Pattern 2 *v*. Pattern 1 were older, fewer practised structured PA and had a lower TPA, greater sedentary behaviour, lower diet quality and later caloric midpoint, while both presented similar EI patterns and weight status (Table 3). The proportion of participants with PA reports on summer holidays was higher for PA Pattern 2 (*P* < 0·001) (online Supplementary Table S6).

**Table 3.** Participants’ characteristics according to temporal physical activity (PA) patterns (*n* 595) (Numbers and percentages; mean values and standard deviations; median values and interquartile ranges)

	PA Pattern 1 – Late morning, mid-afternoon and early evening		PA Pattern 2 – Late afternoon		
	*n* 381		*n* 214		
	*n* mean or median*	% sd or IQR*	*n* mean or median*	% sd or IQR*	*P* †
Sex					0·42
Female	200	52·5	105	49·1	
Male	181	47·5	109	50·9	
Age, years	9·8	2·68	10·4	2·66	0·008
Age group					0·14
Children	164	43·0	79	36·9	
Adolescents	217	57·0	135	63·1	
Maternal age, years‡	40·0	5·15	41·4	5·01	0·002
Parental education‡					0·62
Secondary or lower	228	60·5	141	66·5	
Tertiary	149	39·5	71	33·5	
Maternal work status‡					0·62
Employed	310	81·8	176	83·4	
Unemployed or other	69	18·2	35	16·6	
zBMI, sd	0·66	1·09	0·70	1·12	0·69
Weight status					0·99
Underweight or normal weight	242	63·5	136	63·6	
Overweight or obesity	139	36·5	78	36·4	
Temporal EI patterns					0·74
EI Pattern 1 – Early afternoon and early evening	72	18·9	35	16·4	
EI Pattern 2 – Early afternoon and late evening	270	70·9	156	72·9	
EI Pattern 3 – Late morning, early and mid-afternoon and Late evening	39	10·2	23	10·7	
Healthy eating index score, points	20·7	3·28	19·8	3·52	0·001
TEI, kJ	7937	2218·8	7950	2067·7	0·95
Protein, %TEI	17·1	3·26	17·0	3·33	0·72
Carbohydrates, %TEI	51·0	6·24	51·3	5·89	0·61
Free sugars, %TEI	11·8	5·86	13·0	5·89	0·02
Fibre, g/d§	15·2	7·24	15·2	5·68	0·24
Fat, %TEI	30·0	5·78	30·0	5·66	0·91
Trans fatty acids, %TEI§	0·4	0·31	0·5	0·31	0·38
Saturated fatty acids, %TEI	10·8	2·51	10·7	2·86	0·62
Daily frequency of eating occasions, *n*/d	6·2	1·25	6·1	1·49	
Breakfast-skipping					0·004
No	361	94·8	189	88·3	
Yes	20	5·2	25	11·7	
Breakfast, h:min§	8:22	1:12	8:37	1:55	0·004
Lunch, h:min§	12:52	0:45	13:00	0:51	0·002
Dinner, h:min§	20:15	0:42	20:22	0:52	0·04
First EI occasion, h:min§	8:30	1:22	8:30	2:00	0·31
25 % of TEI, h:min§	11:47	1:52	12:12	1:52	0·01
50 % of TEI, h:min§	14:40	2:22	14:45	2:45	0·05
75 % of TEI, h:min§	19:00	2:00	19:15	2:05	0·11
Last EI occasion, h:min§	20:45	1:15	21:02	1:15	< 0·001
Eating midpoint, h:min§ ^,║^	14:41	1:01	14:53	1:15	0·006
Structured PA‡					0·004
No	127	34·2	98	46·2	
Yes	244	65·8	114	53·8	
Total daily physical activity intensity (TPA), MET-h/d§	21·5	8·28	22·9	8·40	0·03
Time spent in moderate to vigorous intensity PA, h:min	1:32	1:12	1:44	1:27	0·08
Time spent in sedentary behaviour, h:min	6:33	1:53	6:57	2:21	0·03
First PA occasion, h:min§	08:22	1:05	08:38	1:38	0·004
25 % of TPA, h:min§	11:56	1:01	12:38	1:24	< 0·001
50 % of TPA, h:min§	15:16	1:09	15:56	1:05	< 0·001
75 % of TPA, h:min§	18:24	0:58	18:35	1:15	< 0·001
Last PA occasion, h:min§	21:48	0:56	22:08	1:05	< 0·001
Midpoint of PA time window, h:min§	15:04	0:47	15:12	1:10	0·001
Sleep start weekdays, h:min§	22:00	0:57	22:27	1:11	< 0·001
Sleep end weekdays, h:min§	07:35	1:00	08:00	2:22	< 0·001
Sleep duration weekdays, h:min§	09:45	1:15	09:48	1:40	0·42
Sleep start weekends, h:min	22:30	1:15	23:00	1:49	< 0·001
Sleep end weekends, h:min	09:05	1:01	09:58	1:16	< 0·001
Sleep duration weekends, h:min	10:34	1:09	10:56	1:28	0·001
Midpoint of sleep on free days corrected (MSFsc), h:min	03:26	0:48	04:03	1:11	< 0·001

IQR, interquartile range; zBMI, z-score BMI; MET, metabolic equivalent of tasks; TEI, total daily energy intake; EI, energy intake.

* Data are presented as frequency (*n*) and column proportions (%) for categorical variables, as mean and standard deviation (sd) for continuous variables or as the median and interquartile range, if these were non-normally distributed.

† *P*-value for Pearson’s *χ* ^2^ test for categorical variables and Student’s *t* test or Mann–Whitney *U* test for continuous variables.

‡ Maternal age based on *n* 514, due to missing data. Sleep on weekends and MSFsc-related variables based on *n* 591 due to missing data. For categorical variables, the sum of each category did not add up to the *n* of each column due to missing data.

§ Variables for which median (IQR) and *P*-value for Mann–Whitney *U* test are presented.

║ Calculated as (clock time of last EI occasion – clock time of first EI occasion)/2.

### Association between temporal energy intake and physical activity patterns and BMI category

EI Pattern 3 – ‘Late morning, early and mid-afternoon and late evening’ *v*. Pattern 1 – ‘Early afternoon and early evening’, was negatively associated with having overweight or obesity (zBMI ≥ +1 sd) even after adjustments (model 4: OR = 0·49; 95 % CI 0·26, 0·92).

PA Pattern 1 *v*. Pattern 2 – ‘Late afternoon’ was not associated with overweight or obesity in crude and adjusted models, even for MSFsc (model 5: OR = 0·93; 95 % CI 0·65, 1·34) (Table 4).

**Table 4.** Associations of temporal energy intake (EI) patterns and physical activity (PA) patterns with weight status (underweight or normal weight *v*. overweight or obesity) by ordinal logistic regression analysis (Odds ratios and 95 % confidence intervals)

	Overweight or obesity†									
	Model 1 (Crude)‡		Model 2‡		Model 3‡		Model 4‡,§		Model 5||	
Independent variables	OR	95 % CI lower bound, 95 % CI upper bound	OR	95 % CI lower bound, 95 % CI upper bound	OR	95 % CI lower bound, 95 % CI upper bound	OR	95 % CI lower bound, 95 % CI upper bound	OR	95 % CI lower bound, 95 % CI upper bound
Temporal EI patterns (*n* 714)*										NA
EI Pattern 1 – Early afternoon and early evening (*n* 144)	1		1		1		1			
EI Pattern 2 – Early afternoon and late evening (*n* 498)	0·77	0·52, 1·11	0·77	0·53, 1·12	0·77	0·53, 1·13	0·79	0·54, 1·16		
EI Pattern 3 – Late morning, early and mid-afternoon and late evening (*n* 72)	0·48	0·26, 0·89	0·48	0·26, 0·88	0·46	0·25, 0·86	0·49	0·26, 0·92		
Temporal PA patterns (*n* 595)*										
PA Pattern 1 – Late morning, mid-afternoon and early evening (*n* 381)	1		1		1		1		1	
PA Pattern 2 – Late afternoon (*n* 214)	1	0·71, 1·41	0·99	0·7, 1·36	0·95	0·67, 1·36	0·93	0·65, 1·34	0·95	0·65, 1·38

NA, not applicable, due to missing data on the covariate midpoint of sleep on free days corrected for sleep debt (MSFsc).

* For analyses with EI and PA patterns as independent variables, Pattern 1 was the reference category; thus OR = 1. The sample size of each model may not add up to 714 for EI patterns or 595 for PA patterns, due to missing data on adjustment covariates.

† The outcome was modelled with *overweight or obesity* as the response.

‡ Models included the following variables (and sample size), respectively, for EI and PA patterns: *model 1*: EI/PA patterns (*n* 714/*n* 595); *model 2:* model 1 adjusted for sex, age and parental education (*n* 707/ *n* 589); *model 3*: model 2 plus healthy eating index (*n* 707/ *n* 589); *model 4* for EI patterns: model 3 plus structured PA (*n* 691).

§ *Model 4* for PA patterns: model 3 plus time spent in sedentary behaviour (*n* 585).

|| *Model 5* for PA patterns: model 4 plus MSFsc (*n* 574).

## Discussion

### Temporal energy intake patterns

In our study, three EI patterns emerged and Pattern 3 – ‘Late morning, early and mid-afternoon and late evening’ was negatively associated with having overweight or obesity, compared with Pattern 1 – ‘Early afternoon and early evening’. EI Pattern 3, had more peaks, all providing a lower %TEI, than those of Patterns 1 and 2 – ‘Early afternoon and late evening’. Additionally, Pattern 3 had a higher %TEI ingested in the mid-morning and mid-afternoon, contributing to an earlier achievement of 50 % of TEI, that is, caloric midpoint^(41)^, and 75 % of TEI. Therefore, Pattern 3 could be interpreted as having a relatively earlier overall distribution of TEI, and it was associated with a lower risk of having overweight or obesity, compared with Pattern 1.

Although mechanisms are not fully clear, concentrating EI towards later in the day may be associated with adiposity due to the mismatch between the fasting-feeding and light-dark cycles and the biological clocks of the circadian system (chrono-disruption)^(6)^. In turn, metabolic processes and hormonal levels may become dysregulated, leading to an increased appetite, especially for highly palatable foods, insulin resistance, lower energy expenditure and fat accumulation^(6,42,43)^. Biologically, food intake synchronises molecular peripheral clocks ubiquitous in the body, such as in the pancreas and muscles. These clocks intercommunicate with the central hypothalamic clock, the suprachiasmatic nuclei, via neuro-humoral signals. Peripheral clocks are implicated in crucial physiological processes, including the secretion of insulin and hormones involved in energy and nutrient metabolism, appetite and the reward system^(6)^. Behaviourally, individuals with a later EI concentration may exhibit obesity-related behaviours, such as a greater screen time, breakfast-skipping and a lower diet quality, as well as a later chronotype or bedtimes^(44)^ and shorter sleep duration^(7)^ which we did not observe in our study.

Previous epidemiological studies have reported inconsistent results on the association between later eating rhythms and overweight or obesity, in children^(10,45–49)^ and in adults^(8,9,50)^. The divergency of results may be affected by methodological heterogeneity in the assessment methods and in the criteria to define eating rhythms as ‘later’, which encompasses mealtimes^(47)^, the absolute EI or the %TEI ingested at different meals^(49)^, at time-intervals^(45,48)^ or after clock time cut-offs^(46)^. Since few works in children have focused on time-based EI patterns, studies with related exposures will be discussed, although they are not directly comparable.

In adults, a cross-sectional study with a similar analysis found that participants with a pattern marked by consistent and moderate peaks exhibited a lower risk of obesity^(15)^. The %TEI ingested after 20:00 was cross-sectionally positively associated with a higher BMI in children and adolescents^(46)^. In young adults, having a ‘later’ caloric midpoint (≥ 15:30, the sample’s median) was associated with a higher BMI^(50)^. In a national UK survey, adolescents with an EI pattern marked by earlier main meals and night snacks, compared with slightly later main meals but no night snacks, had higher BMI^(47)^. As the results for mealtimes and %TEI across time may differ, the importance of analysing both parameters for main meals and snacks is highlighted.

Results for EI Pattern 3 *v*. Pattern 1 are in line with those of studies focusing on EI Patterns characterised by timing, magnitude and frequency of %TEI peaks^(15)^ or on single and less detailed measures of the temporal daily distribution of %TEI^(46,50)^. EI Pattern 3 had more and even peaks of %TEI occurring during earlier periods within the daytime, which contributed to an earlier caloric midpoint, compared with Pattern 1. The distribution of EI of Pattern 3 was also more even across all 2-h intervals, compared with Patterns 1 and 2, for which the magnitude of peaks diverged more, which is in line with a previous work ^(15)^. However, if only the times of main meals were being compared across patterns, which were later for EI Pattern 3 *v.* Pattern 1, our findings would disagree with works that suggested later mealtimes were linked to higher adiposity^(47)^.

EI Pattern 2 was not associated with having overweight or obesity compared with Patterns 1 or 3 (data not shown). EI Patterns 2 and 1 had a similar magnitude of %TEI peaks, while Patterns 2 and 3 had a similar caloric midpoint and time of last EI occasion, which might have contributed to the null association. Likewise, some works found no significant associations of EI patterns, defined by the %TEI within 2*–*3-h intervals across 06:00 to 24:00^(48)^ or by the %TEI at different meals^(49)^ with BMI, in 2- to 16-year-olds.

Additionally, the %TEI at each 2-h interval should not be mistaken for meals. In EI Patterns 1 and 2, peaks coincided mainly with the reported times of lunch and dinner. However, for Pattern 3, which exhibits a higher variance in data, the first three peaks could coincide either with breakfast or lunch times. This reinforces the relevance of analysing the distribution of %TEI across the 24-h day besides mealtimes.

### Temporal physical activity patterns

Two PA patterns were identified. Although PA Pattern 2 – ‘Late afternoon’, was marked by a concentration of PA later in the day, compared with PA Pattern 1 – ‘Late morning, mid-afternoon and early evening’, no evidence of an association between these PA patterns and weight status was found.

The endogenous response induced by PA, including in the metabolism of energy and nutrients, food intake and sleep, may vary across the 24-h day^(11)^, although evidence from studies in adults is still conflicting and lacking in paediatric age. While a lower percentage of activity counts before noon was cross-sectionally associated with a higher BMI^(12)^, a study that applied a cluster analysis found that a PA pattern with higher activity counts performed throughout the day, early (08:00–11:00) or late (16:00–21:00), exhibited lower BMI compared with patterns with lower activity counts^(16)^. Comparison with our results is hindered by the different age ranges and methodological heterogeneity to derive temporal PA clusters, as we used PA diaries to estimate the %TPA within 2-h intervals, whereas previous research used accelerometers to assess the absolute PA counts during the morning/evening periods^(12,16)^.

We obtained only two clusters, and none was a ‘morning’ pattern, contrasting to the previous studies in adults. The timing of PA of 6- to 14-year-olds is mostly influenced by school schedules^(31)^, and differences in PA patterns may become more noticeable in older individuals. The reduced variability of patterns and a lower prevalence of overweight or obesity in this sub-sample (*v*. individuals without PA diaries) may have contributed to the null association. Nonetheless, participants with PA Pattern 2 presented a clustering of overweight-related behaviours (lower diet quality, higher sedentarism and later sleep times), previously reported since a young age^(51)^ and, thus, should be investigated to help identify risk factors for obesity.

### Strengths and limitations

Strengths of this study include the harmonised methodology to collect dietary intake by trained staff with a background in nutrition sciences or dietetics^(19)^, and the classification of earlier/later patterns without criteria based on mealtimes or cut-off definitions, in an understudied population.

Limitations include possible measurement errors and social desirability bias of dietary intake, including differential under/overreporting according to meals or weight status^(47,52)^. Also, we used PA diaries to estimate PA intensity, which is less accurate than objective methods.

Cluster misclassification of individuals is a possible limitation. Two dietary intake/three PA reports may be insufficient to accurately measure the temporal daily distribution of EI and PA due to intraindividual variability^(53)^. Each eating occasion was allocated to a single 2-h interval, although the reported clock time could have been close to two consecutive intervals contributing to the %TEI at one interval *v*. the other. Nevertheless, we assessed %TEI differences between every 2-h interval within each pattern, to ensure similar peaks would be considered.

EI patterns were not associated with different types of days of dietary intake reports presented by participants. Although EI Pattern 2 *v*. Pattern 1 was more prevalent in participant’s filling reports during the summer holidays, there was no difference between EI Patterns 1 and 3.

Additionally, participants with dietary reports and PA diaries on summer holidays *v*. other periods had similar characteristics, including on covariates and weight status, although the sample size was reduced for some comparisons. Therefore, we do not expect this to have biased our association results.

Moreover, our results should be interpreted with caution given the sample size of Pattern 3, due to a higher variance of data and a lower precision of the estimated association.

Given the cluster approach to derive temporal EI and PA patterns, one cannot disentangle the exact factors that explain their association with weight status, for example, the consistency of the magnitude of %TEI/%TPA peaks, the temporal distribution of peaks, or the %TEI/TPA attained at a specific time cut-off. Nevertheless, we discussed the plausibility and postulated explanations for our findings, considering current knowledge.

We cannot exclude the potential role of unmeasured covariates or those with high missingness in our results, such as pubertal stage, chronotype and sleep in EI patterns’ analyses. The sub-sample of PA patterns’ analyses had a lower prevalence of overweight or obesity compared with excluded individuals, limiting the generalisability of results.

Lastly, the cross-sectional study design does not allow the establishment of a causal association between patterns and BMI within this population.

Therefore, future research on temporal patterns should include larger samples with older adolescents, a higher number of observations during weekly and annual variations, and explore jointed patterns of EI and PA applying accelerometry^(9)^.

### Conclusion

Having a temporal EI pattern with more and even peaks of proportions of TEI throughout the day, mostly concentrated at earlier daytime periods, compared with less and higher peaks at later times, may be associated with a lower risk of overweight and obesity, in Portuguese youth aged 6–14 years. In contrast, the identified temporal PA patterns do not appear to be linked with body weight status. Investigating the distribution of caloric intake and PA intensity across the 24-h day, besides clock times of main meals, and the associated overweight-related behaviours may be important to identify risk factors for paediatric obesity.

## Supporting information

Cardoso et al. supplementary material Cardoso et al. supplementary material

## Supplementary material

For supplementary material/s referred to in this article, please visit https://doi.org/10.1017/S0007114524002861.
