# Supplementary material for: Temporal patterns of energy intake and physical activity and cross-sectional associations with body weight status in children and adolescents: results from the Portuguese National Food, Nutrition and Physical Activity Survey 2015–2016
Source: Br J Nutr. 2024 Nov 11;132(12):1684–97. doi: 10.1017/S0007114524002861 (PMC11695112; doi:10.1017/S0007114524002861)
Supplement: Cardoso et al. supplementary material [file S0007114524002861sup001.docx]

**SUPPLEMENTARY MATERIAL** for the manuscript “Temporal patterns of energy intake and physical activity and cross-sectional associations with body weight status in children and adolescents: results from the Portuguese National Food, Nutrition and Physical Activity Survey 2015–2016” by Sofia Cardoso, Inês Sanches, Daniela Correia, Sofia Vilela

**Table S1**. Daily average proportion of total daily energy intake (%TEI) ingested at each 2-h interval of the 24-h day, according to Temporal Energy Intake (EI) patterns (n=714).

|  | EI Pattern 1 – Early afternoon & Early evening | | EI Pattern 2 – Early afternoon & Late evening | | EI Pattern 3 – Late morning, Early and Mid-afternoon & Late evening | |  |
| --- | --- | --- | --- | --- | --- | --- | --- |
|  | *n* 144 | | *n* 498 | | *n* 72 | |  |
|  | **% TEI** | | **% TEI** | | **% TEI** | |  |
| 2-h interval (local clock times in h:min) | **Median*** | **IQR*** | **Median*** | **IQR*** | **Median*** | **IQR*** | **p-value**^†^ |
| [00:00,02:00) | 0 | 0 | 0 | 0 | 0 | 0 | 0.09 |
| [02:00,04:00) | 0 | 0 | 0 | 0 | 0 | 0 | 0.75 |
| [04:00,06:00) | 0 | 0 | 0 | 0 | 0 | 0 | 0.33 |
| [06:00,08:00) | 0 | 9.569 | 0 | 8.769 | 0 | 7.641 | 0.46 |
| [08:00,10:00) | 6.64 | 11.315 | 6.77 | 13.494 | 6.12 | 10.252 | 0.276 |
| [10:00,12:00) | 8.05 | 10.645 | 9.70 | 9.909 | 14.21 | 16.389 | <0.001 |
| [12:00,14:00) | 24.39 | 8.998 | 26.48 | 11.787 | 15.97 | 15.362 | <0.001 |
| [14:00,16:00) | 1.20 | 9.539 | 0 | 8.575 | 18.63 | 14.865 | <0.001 |
| [16:00,18:00) | 10.97 | 13.967 | 12.89 | 11.672 | 9.28 | 12.626 | 0.04 |
| [18:00,20:00) | 25.28 | 12.111 | 3.59 | 10.085 | 3.99 | 10.667 | <0.001 |
| [20:00,22:00) | 6.32 | 12.251 | 24.00 | 12.741 | 15.81 | 10.171 | <0.001 |
| [22:00,24:00) | 0 | 5.677 | 0 | 2.843 | 0 | 6.470 | 0.02 |

Abbreviations: n, frequency (sample size of each pattern); %, proportion; TEI, total daily energy intake; IQR, interquartile range.

* Data are presented as the median and interquartile range (IQR), as continuous variables were non-normally distributed.

† P-value for Independent-Samples Kruskal-Wallis test.

**Table S2**. Participants’ characteristics regarding PA-related covariates, according to Temporal Energy Intake (EI) patterns, for the sub-sample included in PA patterns’ analyses (n=595).

|  | **EI Pattern 1** – **Early afternoon & Early evening** | | **EI Pattern 2** – **Early afternoon & Late evening** | | **EI Pattern 3** – **Late morning, Early and Mid-afternoon & Late evening** | |  |
| --- | --- | --- | --- | --- | --- | --- | --- |
|  | *n* 107 | | *n* 426 | | *n* 62 | |  |
|  | **Median**^*^ | **IQR**^*^ | **Median**^*^ | **IQR**^*^ | **Median**^*^ | **IQR**^*^ | **p-value**^†^ |
| Total Daily Physical activity intensity (TPA), METs/d | 20.8 | 9.32 | 22.6 | 8.43 | 21.1 | 8.64 | 0.10 |
| Time spent in Moderate to vigorous intensity PA, h:min | 1:22 | 1:46 | 1:23 | 1:41 | 0:55 | 1:43 | 0.26 |
| Time spent in Sedentary behaviour, h:min | 6:10 | 2:41 | 6:25 | 2:43 | 6:38 | 2:46 | 0.42 |
| First PA occasion^,^ h:min | 8:18 | 1:05 | 8:30 | 1:16 | 8:33 | 1:03 | 0.08 |
| 25% of TPA, h:min | 12:01 | 1:09 | 12:11 | 1:18 | 12:11 | 1:07 | 0.12 |
| 50% of TPA, h:min | 15:22 | 1:13 | 15:35 | 1:13 | 15:31 | 1:07 | 0.21 |
| 75% of TPA, h:min | 18:15 | 1:11 | 18:28 | 1:00 | 18:24 | 1:16 | 0.05 |
| Last PA occasion, h:min | 21:41 | 1:09 | 21:58 | 0:54 | 22:03 | 1:20 | 0.02 |
| Midpoint of PA window, h:min | 14:56 | 0:46 | 15:10 | 0:56 | 15:08 | 0:50 | 0.006 |
| Sleep start weekdays, h:min | 21:45 | 1:15 | 22:10 | 1:00 | 22:10 | 1:05 | 0.001 |
| Sleep end weekdays, h:min | 7:30 | 1:00 | 7:45 | 1:37 | 7:52 | 1:45 | 0.02 |
| Sleep duration weekdays, h:min | 9:50 | 1:10 | 9:45 | 1:25 | 9:46 | 1:42 | 0.89 |
| Sleep start weekends, h:min^‡^ | 22:15 | 1:30 | 22:30 | 1:30 | 22:45 | 1:45 | 0.11 |
| Sleep end weekends, h:min^‡^ | 9:07 | 1:37 | 9:22 | 1:37 | 9:15 | 1:15 | 0.28 |
| Sleep duration weekends, h:min^‡^ | 10:39 | 1:22 | 10:37 | 1:37 | 10:37 | 1:30 | 0.85 |
| MSFsc, h:min^‡^ | 3:22 | 0:54 | 3:36 | 1:16 | 3:25 | 1:19 | 0.07 |

Abbreviations: n, frequency (sample size of each pattern); %, proportion; IQR, interquartile range; PA, physical activity; TPA, total daily physical activity intensity; METs, metabolic equivalent of tasks; d, day; h, hours; min, minutes, MSFsc, Midpoint of sleep on free days corrected.

* Data are presented as the median and interquartile range (IQR), as continuous variables were non-normally distributed.

† P-value for Independent-Samples Kruskal-Wallis test.

^‡^ Based on n=107, n=423, n=61, for EI Patterns 1, 2, and 3, respectively, due to missing data.

## **Table S3.** Daily average clock times of EI parameters, not weighted and weighted by the type of day of the week, for participants with dietary intake reports on one weekday and one weekend day (n=169).

|  | Participants with dietary intake reports on one weekday and one weekend day | | | |
| --- | --- | --- | --- | --- |
|  | *n* 169 | | *n* 169 | |
|  | Not weighted daily average^*^ | | Weighted daily average^†^ | |
| Clock times of EI parameters | **Median^‡^** | **IQR^‡^** | **Median^‡^** | **IQR^‡^** |
| First EI occasion^,^ h:min | 8:55 | 1:03 | 8:38 | 1:17 |
| 25% of TEI, h:min | 12:15 | 1:57 | 12:15 | 2:06 |
| 50% of TEI, h:min | 14:45 | 2:30 | 14:57 | 2:43 |
| 75% of TEI, h:min | 19:00 | 2:00 | 19:11 | 2:22 |
| Last EI occasion, h:min | 21:00 | 1:06 | 21:00 | 1:11 |
| Eating midpoint, h:min^§^ | 15:02 | 0:56 | 14:51 | 0:55 |

Abbreviations: h, hours; IQR, interquartile range; min, minutes; n, frequency (sample size); TEI, Total daily energy intake.

* Calculated as (clock time of EI parameter on the weekday + clock time of EI parameter on the weekend day) / 2.

† Calculated as [(clock time of EI parameter on the weekday $\times$5) + (clock time of EI parameter on the weekend day$\times$ 2) / 7].

‡ Data are presented as the median and interquartile range (IQR), as continuous variables were non-normally distributed.

§ Eating midpoint is the midpoint of the eating window and was calculated as (clock time of last EI occasion – clock time of first EI occasion) / 2*.*

## **Table S4.** Association between participants’ period of dietary intake reports and Temporal Energy Intake (EI) patterns (n=714).

|  | EI Pattern 1 – Early afternoon & Early evening | | EI Pattern 2 – Early afternoon & Late evening | | EI Pattern 3 – Late morning, Early and Mid-afternoon & Late evening | |  |
| --- | --- | --- | --- | --- | --- | --- | --- |
|  | *n* 144 | | *n* 498 | | *n* 72 | |  |
|  | ***n**** | **%*** | ***n**** | **%*** | ***n**** | **%*** | **p-value**^†^ |
| Participants’ period of dietary intake reports^‡^ |  |  |  |  |  |  | 0.006 |
| Summer school holidays | 27 | 18.8^a^ | 162 | 32.5^b^ | 20 | 27.8^a,b^ |  |
| Other | 117 | 81.3^a^ | 336 | 67.4^b^ | 52 | 72.2^a,b^ |  |

Abbreviations: n, frequency; %, proportion, EI Energy intake.

Following a z-test for column proportions Bonferroni-adjusted, considering p-values, different superscripts are attributed to the patterns with different proportions.

* Data are presented as frequency of participants (n) and column proportions (%).

† P-value for Pearson’s Chi-square test.

‡ Each participant’s period of dietary intake reports was categorised as “Summer school holidays” if at least 1 dietary intake report occurred between June 9^th^ and September 15^th^, 2016 (n=209 participants), or as “Other”, if 0 days occurred during that period, which included school terms, Christmas, carnival, and easter holidays (n=505).

**Table S5.** Daily average proportion of total daily physical activity (%TPA) intensity expended at each 2-h interval of the 24-h day, according to Temporal Physical Activity (PA) patterns (n=595).

|  | PA Pattern 1 – Late morning, Mid-afternoon & Early evening | | PA Pattern 2 – Late-afternoon | |  |
| --- | --- | --- | --- | --- | --- |
|  | *n 381* | | *n 214* | |  |
|  | **% TPA** | | **% TPA** | |  |
| 2-h interval (local clock times in h:min) | **Median*** | **IQR*** | **Median*** | **IQR*** | **p-value**^†^ |
| [00:00,02:00) | 0 | 0 | 0 | 0.330 | <0.001 |
| [02:00,04:00) | 0 | 0 | 0 | 0 | <0.001 |
| [04:00,06:00) | 0 | 0 | 0 | 0 | 0.26 |
| [06:00,08:00) | 1.61 | 3.072 | 0.56 | 2.539 | <0.001 |
| [08:00,10:00) | 8.44 | 4.819 | 5.80 | 5.840 | <0.001 |
| [10:00,12:00) | 15.37 | 5.047 | 10.76 | 5.873 | <0.001 |
| [12:00,14:00) | 12.85 | 4.622 | 13.83 | 5.306 | 0.002 |
| [14:00,16:00) | 15.77 | 4.901 | 12.75 | 5.244 | <0.001 |
| [16:00,18:00) | 13.57 | 4.401 | 21.33 | 7.006 | <0.001 |
| [18:00,20:00) | 16.49 | 6.112 | 13.90 | 5.263 | <0.001 |
| [20:00,22:00) | 11.03 | 3.999 | 11.39 | 4.717 | 0.24 |
| [22:00,24:00) | 1.72 | 3.946 | 3.98 | 7.351 | <0.001 |

Abbreviations: n, frequency (sample size of each pattern); %, proportion; TPA, total daily physical activity intensity; h, hour; min, minutes; IQR, interquartile range.

* Data are presented as the median and interquartile range (IQR), as continuous variables were non-normally distributed.

† P-value for Independent-Samples Mann-Whitney U test.

## **Table S6.** Association between participants’ period of PA diaries and Temporal Physical Activity (PA) patterns (n=595).

.

|  | PA Pattern 1 – Late morning, Mid-afternoon & Early | | PA Pattern 2 – Late afternoon | |  |
| --- | --- | --- | --- | --- | --- |
|  | *n 381* | | *n 214* | |  |
|  | ***n**** | **%*** | ***n**** | **%*** | **p-value**^†^ |
| Participants’ period of PA diaries^‡^ |  |  |  |  | <0.001 |
| Summer school holidays | 91 | 24.0 | 82 | 38.3 |  |
| Other | 288 | 76.0 | 132 | 61.7 |  |

Abbreviations: n, frequency of participants; %, proportion; PA, physical activity.

* Data are presented as frequency of participants (n) and column proportions (%).

† P-value for Pearson’s Chi-square test.

‡ Each participant’s period of PA diaries was categorised as “Summer school holidays” if at least 1 PA diary report occurred between June 9^th^ and September 15^th^, 2016 (n=173 participants), or as “Other”, if 0 days occurred during that period, which included school terms, Christmas, carnival, and easter holidays (n=422). Additionally, we confirmed that all participants categorised as “Summer school holidays” reported all PA diaries during this period.
